# LIP expression is regulated by IGF-1R signaling and participates in suppression of anoikis

**DOI:** 10.1186/1476-4598-10-100

**Published:** 2011-08-19

**Authors:** Huili Li, Brenda R Baldwin, Cynthia A Zahnow

**Affiliations:** 1 Department of Oncology, the Sidney Kimmel Comprehensive Cancer Center at Johns Hopkins, Baltimore Maryland, 21231, USA; 2 FDA/CBER, 1401 Rockville Pike, Rockville, MD, 20852-1448, USA

**Keywords:** C/EBPβ, LIP/LAP, IGF-1R, EGFR, Breast Cancer, MCF10A, Anoikis

## Abstract

**Background:**

The transcription factor, CCAAT enhancer binding protein-β (C/EBPβ), is expressed as several distinct protein isoforms (LAP1, LAP2 and LIP) that have opposing actions in cellular proliferation and differentiation. Increases in the ratio of LIP/LAP are associated with aggressive, metastatic breast cancer; however, little is known regarding the molecular mechanisms that regulate LIP expression or the biological actions of an increase in the LIP/LAP ratio. Metastasis is highly dependent upon the suppression of anoikis and the role of C/EBPβ and LIP in this anchorage-independent, survival process is currently not known in mammary epithelial cells. IGF-1R signaling is important for the survival of breast cancer cells and crosstalk between IGF-1R and EGFR signaling pathways have been implicated in the development of more aggressive disease. We therefore evaluated in mammary epithelial cells whether IGF-1R signaling regulates the LIP/LAP ratio, analyzed the potential interplay between EGFR and IGF-1R signaling and addressed the biological significance of increased LIP expression in cellular survival and suppression of anoikis.

**Results:**

Our data provide the first evidence that IGF-1R signaling regulates LIP expression in an EGFR independent manner to increase the LIP/LAP ratio in mammary epithelial cells. Although crosstalk between IGF-1R signaling and EGFR signaling is detectable in MCF10A cells, this crosstalk is not required for the IGF-1 mediated regulation of LIP expression. Rather, the critical regulator of IGF-1 induced LIP expression appears to be EGFR-independent, Akt activity. Our data also demonstrate that increases in LIP expression promote cell survival via suppression of anoikis. Likewise, knockdown of total C/EBPβ leads to increased cell death and suggest that C/EBPβ expression is important for survival and resistance to anoikis. IGF-1 treatment can partially rescue vector control cells from anoikis; however, cells with reduced C/EBPβ expression do not survive anoikis.

**Conclusions:**

Taken together, our data demonstrate that IGF-1R signaling regulates LIP expression in an EGFR independent manner to increase the LIP/LAP ratio in mammary epithelial cells. C/EBPβ expression and elevations in LIP play an important role in regulating cellular survival via suppression of anoikis, in an IGF-1R mediated context or in a manner independent of IGF-1R signaling.

## Background

The transcription factor, CCAAT/Enhancer binding protein β (C/EBPβ) is an important mediator of mammary development [1,2] and breast tumorigenesis [3,4]. Encoded by an intronless gene, C/EBPβ is expressed as several distinct protein isoforms (LAP1, LAP2 and LIP) whose expression is tightly regulated by the differential use of a number of in-frame translation start sites [5-7]. All of the C/EBPβ isoforms share the same C-terminal DNA binding and leucine zipper dimerization domains, but LIP lacks all of the N-terminal transactivation domain and much of the inhibitory domains. Consequently, LIP can act as a dominant-negative [5] to inhibit gene transcription or as an activator of transcription, depending upon the nature of its interaction with other C/EBP family members and transcription factors [8-11].

The LIP and LAP isoforms may thus have potentially opposing actions in cellular proliferation and differentiation and increases in the LIP/LAP ratio are known to be associated with tumorigenesis and metastasis. For example, overexpression of LIP in the rodent mammary gland leads to hyperplasia and tumor formation [12]. In humans, the LIP isoform is strongly expressed in a percentage of aggressive human breast tumors that are estrogen receptor negative, aneuploid, highly proliferative and associated with a poor prognosis [13,14]. In metastatic breast cancer cells, an increase in the LIP/LAP ratio has been linked to a loss in the TGFβ-dependent cytostatic response and a more aggressive phenotype [15]. The C/EBPβ isoforms thereby play an important role in high grade, metastatic breast cancer and the LIP/LAP ratio is a critical determinant in the aggressiveness of the disease.

It is therefore imperative, that we better understand the molecular mechanisms regulating LIP expression and the biological significance of the LIP/LAP ratio in breast cancer. Growth factor signaling pathways, such as the insulin-like growth factor-1 receptor (IGF-1R) [16] and the epidermal growth factor receptor (EGFR) signaling cascades [17] have been implicated in the development of aggressive, metastatic breast cancer. IGF-1R signaling contributes to breast cancer progression and recurrence in part by increasing cell survival via mechanisms that include suppression of anoikis [18-21]. Anoikis is an induction of apoptosis that occurs in cells upon loss of cellular adhesion and is one of the hallmarks of metastasis [22]. C/EBPβ has also been shown to play a role in cell survival; specifically, of hepatic cells [23], keratinocytes [24], and macrophages [25], but has not yet been associated with suppression of anoikis. Moreover, it is also not known whether LIP plays a specific role to increase the survival of breast cancer cells. To better understand the molecular mechanisms that regulate LIP expression in metastatic breast cancer, we set out to determine in mammary epithelial cells whether IGF-1R signaling leads to an increase in LIP expression and whether LIP plays a role in IGF-1R mediated suppression of anoikis.

Numerous studies have demonstrated that the actions of IGF-1R are linked to that of EGFR in epithelial mammary cells to synergistically drive cellular proliferation [26-30]. Additional reports have characterized a relationship between IGF-1R and EGFR signaling in aggressive, drug-resistant breast cancer cells and have speculated that IGF-1R signaling plays a role in the development of gefitinib resistant EGFR tumors [31]. Because our previous study [32], demonstrated that LIP expression is increased by EGFR signaling, this led us to question, and to address in this study whether IGF-1R signaling can solely regulate LIP expression and whether crosstalk and activation of the EGF receptor is required. Along these lines, a recent study showed how changes in the LIP/LAP ratio downstream of HER2 provide evasion to oncogene induced senescence and TGFβ cytostasis [33]. These authors showed that changes in LIP/LAP ratio, in an AKT dependent manner, support evasion of a tumor suppressor mechanism in metastatic breast cancer cells [33]. Similarly, an earlier study demonstrated that HER2 expression can lead to survival from anoikis in MCF10 and HMEC cells [34].

Our data demonstrate that IGF-1R signaling regulates LIP expression in an EGFR independent manner to increase LIP expression and the LIP/LAP ratio in mammary epithelial cells. Although crosstalk between IGF-1R signaling and EGFR signaling is detectable in MCF10A cells, this crosstalk is not required for the IGF-1 mediated regulation of LIP expression. Rather, the critical regulator of IGF-1 induced LIP expression appears to be EGFR-independent, Akt activity. Our data also demonstrate that a biological action of LIP is to increase cell survival by suppression of anoikis which may occur in either an IGF-1R mediated context or in a manner independent of IGF-1R signaling. Taken together, the accumulated evidence discussed above, as well as our current data suggest that LIP expression may be an important downstream target of EGFR, ErbB2 and IGF-1R signaling in breast cancer.

## Results

### IGF-1R increases the ratio of LIP/LAP expression

**Figure 1:**
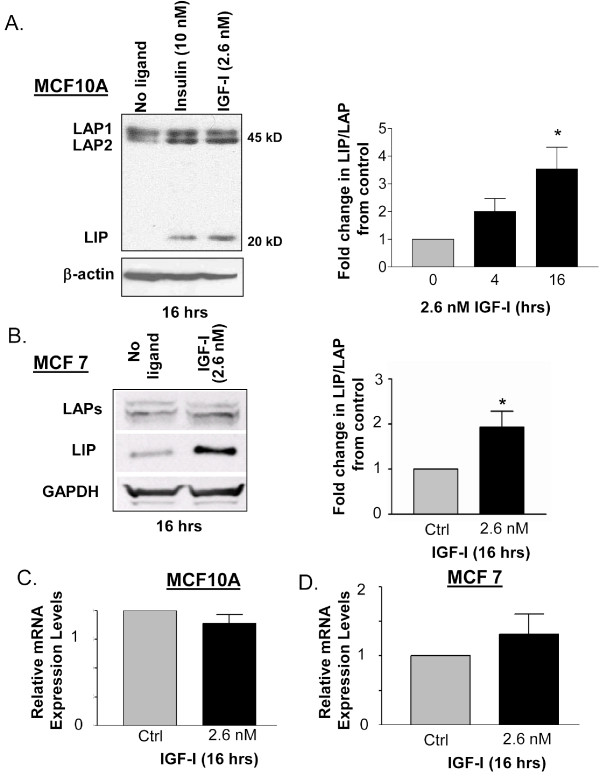
**Stimulation of MCF10A and MCF7 cells with IGF-1 leads to an increase in LIP expression and the LIP/LAP ratio, but not in LIP mRNA expression**. A). Western blot analysis of MCF10A cells serum starved for 24 hours prior to 4, or 16 hours of treatment with IGF-1 (2.6 nM) or insulin (10 nM). Whole cell extracts (100 μg) were analyzed via 12% SDS-PAGE and Western blotting using a monoclonal anti-C/EBPβ antibody. β-actin was used to estimate loading and transfer of proteins. The western blot shown is representative of dozens of separate Western blots. The LIP/LAP ratio was quantitated using Li-COR's Odyssey infrared imaging system. LAP1 and LAP2 expression values were combined and normalized to GAPDH. LIP values were also normalized to GAPDH. The fold change in the LIP/LAP ratio from the IGF-1 treated cells vs. the control cells was calculated and statistical significance was determined via an unpaired, 2-tailed T test with a * p value < 0.05. n = 4. B). Western blot analysis of MCF7 cells serum starved for 24 hours prior to 16 hours of treatment with IGF-1 (2.6 nM). The fold change and statistical significance in the LIP/LAP ratio was studied as Figure 1A (n = 5). C-D). MCF10A and MCF7 cells were treated with IGF-1 for 16 hours. Total RNA was extracted and quantitative realtime PCR was conducted with specific C/EBPβ mRNA primers. No obvious difference was observed in C/EBPβ mRNA expression between untreated and IGF-1 treated cells. (MCF10A; n = 3) (MCF 7; n = 6).

To determine whether IGF-1 regulates C/EBPβ-LIP expression in mammary epithelial cells, MCF10A cells were serum starved for 24 hours and then stimulated with IGF-1 (2.6 nM, 20 ng/ml) for 4 or 16 hours prior to harvesting. Western blot analysis of whole cell extracts demonstrated that treatment with IGF-1 led to an increase in the LIP isoform (Figure. 1A). The LIP isoform was more significantly elevated as compared to the LAP isoforms, resulting in a statistically significant increase in the LIP/LAP ratio of 3.5 fold (p < 0.05) after 16 hrs of treatment as compared to LIP/LAP levels observed in serum starved, non-treated cells (Figure 1A). Similar increases in LIP expression and the LIP/LAP ratio were observed in MCF 7 cells treated with 2.6 nM IGF-1 for 16 hours (Figure 1B). Treatment of cells with insulin (10 nM) also led to increases in LIP protein expression (Figure 1A). The identification and sizes of the human LAP 1 and LAP 2 isoforms were confirmed in our previous study [32].

An IGF-1 concentration of 2.6 nM was chosen for this study because it is within the Kd of the IGF-1 receptor, and will not result in activation of the insulin receptor [35,36]. In some experiments the IGF-1 concentration was increased 15× to 39 nM in order to generate a maximal LIP induction due to activation of IGF-1R, hybrid receptors and the insulin receptor. Likewise an insulin concentration of 10 nM activates insulin receptors but not IGF-1 receptors [35,36]. Because a strong induction in LIP expression was commonly observed 16 hr after IGF-1 treatment, this time point was selected for all consequent analyses in this study.

### IGF-1R does not regulate C/EBPβ mRNA

To determine whether the increase in LIP expression might be the result of transcriptional increases in C/EBPβ mRNA, RNA was purified from IGF-1 (2.6 nM) treated MCF10A and MCF7 cells and C/EBPβ mRNA expression levels were analyzed by real-time qPCR. No statistically significant changes were observed in the levels of C/EBPβ mRNA in response to a 16 hour treatment of cells with 2.6 nM IGF-1 (Figure 1C, D). These data suggest that IGF-1R signaling does not increase C/EBPβ-LIP expression via an increase in C/EBPβ mRNA transcription, but rather via post-transcriptional mechanisms.

### IGF-1R regulates C/EBPβ activity

**Figure 2:**
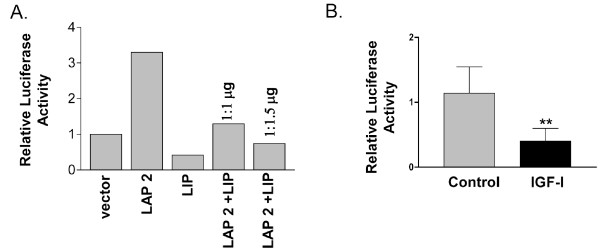
**IGF-1 stimulates the inhibitory effect on a C/EBP consensus construct**. A). To validate the activity of individual LIP and LAP2 constructs, a C/EBP consensus luciferase construct (500 ng) and a Renilla construct (20 ng) as internal control were co-transfected with LAP2 and LIP individually or together at different ratios into C/EBPβ null cells to a total of 2500 ng plasmid DNA. Control vector serves as both a control for basal activity and to match the quantity of plasmid DNA. Luciferase and Renilla activities were recorded at 48 hours. B). IGF-1 stimulation of MCF10A cells regulates C/EBPβ activity. MCF10A cells were transfected with a C/EBP consensus Luciferase construct (500 ng) and a Renilla construct (20 ng) as internal control and serum starved for 24 hours prior to treatment with 39 nM IGF-1. Luciferase activity was analyzed after 16 hours of treatment with IGF-1. The relative luciferase activity was calculated as Luciferase value/Renilla value (n = 5). Statistical significance was determined using an unpaired, 2-tailed T-test with a * p value < 0.05.

It was next important to determine whether the increased expression of LIP and the elevations observed in the LIP/LAP ratio in response to IGF-1 treatment were biologically active. To serve as a control, we first validated the activity of the individual LIP and LAP2 constructs on a C/EBPβ responsive promoter as shown in Figure 2A. C/EBPβ null mammary epithelial cells were transfected with either LIP, or LAP2 individually or together with a C/EBP responsive, firefly luciferase construct and renilla luciferase construct as control. As expected, LAP2 expression led to an increase in C/EBP responsive luciferase activity while LIP alone reduced promoter activity (Figure 2A). In combination with LAP2, LIP expression antagonized and decreased LAP2 induced promoter activity and led to a decrease in luciferase activity. To test for IGF-1 induced, endogenous C/EBPβ activity, MCF10A cells were transfected with a C/EBP responsive, luciferase construct before stimulation with IGF-1. To maximize LIP expression for a significant increase the LIP/LAP ratio, cells were stimulated for 16 hrs with 39 nM IGF-1. This led to an expected decrease in C/EBP responsive luciferase activity due to the antagonistic effects of increased LIP expression (Figure 2B). These data demonstrate that IGF-1R induced increases in the LIP/LAP ratio are biologically active.

### Does IGF-1R and Insulin regulate LIP expression via the activation of the EGF receptor?

**Figure 3:**
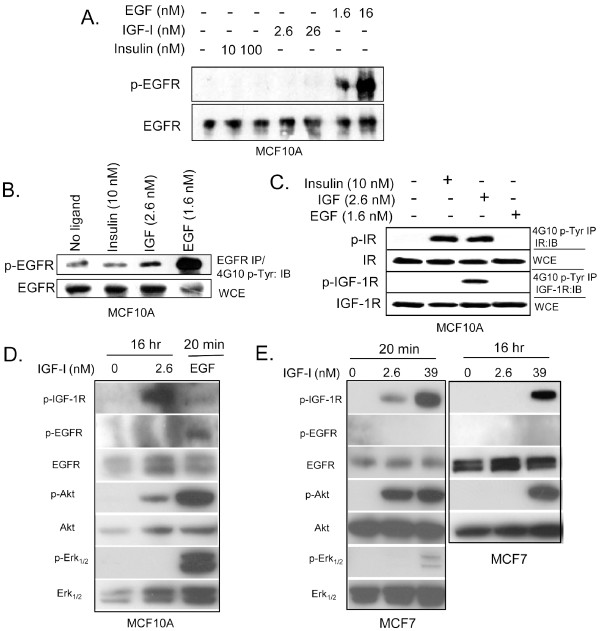
**IGF-1 signaling weakly activates EGFR**. Western blot analysis of serum starved MCF10A and MCF7 cells stimulated with insulin (10 or 100 nM), IGF-1 (2.6 or 26 nM) or EGF (1.6 or 16 nM) for 10-20 minutes or 16 hours and harvested for analysis of phosphorylation status for the EGFR, IGF-1R, insulin receptor, Akt, or Erk1/2. A). MCF10A whole cell protein extracts, 100 μg for phospho-EGFR (p-EGFR) and 50 μg for total EGFR (EGFR) were analyzed via 7% SDS-PAGE and Western Blotting using anti-EGFR antibodies. B). Upper panel: 1-2 mg of whole cell extracts were immunoprecipitated overnight with anti-EGFR, electrophoresed via 7% SDS-PAGE and immunoblotted with anti-phosphotyrosine (4G10). Lower panel: Whole cell extracts (WCE 50 μg) analyzed via 7% SDS-PAGE and Western Blotting using anti-EGFR antibody. C). Whole cell extracts were immunoprecipitated with 4G10 and immunoblotted with anti IGF-1R or anti-insulin receptor. D). Whole cell protein extracts of MCF10A, 100 μg were analyzed via 7% SDS-PAGE and Western Blotting using specific antibodies to p-EGFR, p-IGF-1R, total EGFR (EGFR), p-Akt, p-Erk1/2, total Akt or total Erk1/2. E). Whole cell protein extracts of MCF7, 100 μg were analyzed via 7% SDS-PAGE and Western Blotting using specific antibodies to p-EGFR, p-IGF-1R, total EGFR (EGFR) and p-Akt, p-Erk1/2, total Akt or total Erk1/2.

Because IGF-1R signaling has been observed to cross-talk with EGFR signaling, it was necessary to determine whether the IGF-1R induced expression of LIP was, in part, mediated by EGFR signaling. We therefore investigated whether treatment of MCF10A and MCF7 cells with IGF-1 leads to phosphorylation of EGFR. As determined by Western blot analysis, neither IGF-1 nor insulin stimulation led to a significant increase in EGFR phosphorylation as assessed in whole cell protein extracts 10 minutes (Figure 3A) after addition of ligand. Additionally, neither a 10× increase in IGF-1 nor insulin activated the EGF receptor (Figure 3A). However, immunoprecipitation followed by immunoblot analysis did show a modest increase in phosphorylated EGFR after 10 minutes of IGF-1 stimulation (Figure 3B).

In addition to IGF-1 and insulin receptors, mammary epithelial cells can also express insulin/IGF-1 hybrid receptors [36-38]. Hybrid receptors have been detected in most tissues that express both insulin receptor and IGF-1 receptor. An IGF-1 concentration of 2.6 nM will not activate the insulin receptor, but could potentially lead to the activation of the insulin/IGF 1 hybrid receptors. Data presented in Figure 3C supports this hypothesis and suggests that IGF-1 (2.6 nM) signaling has led to the formation of insulin/IGF-1 hybrid receptors. Functional studies with hybrid receptors demonstrate that they behave more like IGF-1 receptors rather than insulin receptors because they bind IGF-1 with a much better affinity than insulin [37,38]. As expected, we did not observe activation of the hybrid receptor with 10 nM insulin (Figure 3C). Although the significance of the hybrid receptors in mammary epithelial cells in unclear, we hypothesize that the insulin/IGF-1 hybrids may be more abundant in MCF10A cells than otherwise expected and this hypothesis is supported by reports that insulin and hybrid insulin/IGF -1 receptors are important regulators of breast cancer cells [36,38]. Throughout this study, we will refer to the IGF-1R mediated induction in LIP for simplicity, but the reader should understand that hybrid receptors may also be involved in regulation of LIP/LAP.

Because LIP expression is analyzed 16 hr after addition of ligand, we also checked p-EGFR expression at this later time point. EGFR was not phosphorylated in MCF10A cells or MCF 7 cells 16 hr after addition of IGF-1 (Figure 3D, E) To confirm that IGF-1 was indeed activating the IGF-1R signaling cascade, we analyzed p-IGF-1R and p-Akt expression at 20 min and 16 hr (Figure 3D, E).

**Figure 4:**
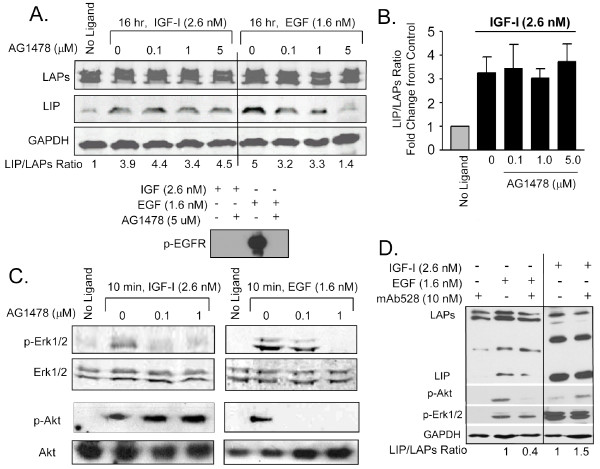
**Pharmacological blockade of EGFR does not inhibit IGF-1 induced increases in LIP expression**. MCF10A cells were serum-starved for 24 hrs, pretreated for 30-60 min with inhibitors, and stimulated with ligands for 10 min or 16 hours. A). Western blot analysis demonstrating that pretreatment of cells with the EGFR inhibitor, AG1478, does not prevent IGF-1R induced LIP expression at 16 hrs, but does antagonize EGF induced LIP expression at doses of 0.1, 1, and 5 μM as compared to control cells stimulated with EGF but without AG1478 treatment (0). The functionality of AG1478 was confirmed by Western blot using a p-EGFR antibody (lower panel). B). LIP/LAPs ratio in MCF10A cells stimulated with IGF-1 and treated with AG1478 as in (A) was quantitated using an Odyssey infrared imaging system. No significant decrease of IGF-1R induced LIP expression or LIP/LAPs ratio was observed with AG1478 treatment at 16 hours as determined via a two-tailed T-test (n = 3). C). Western blot demonstrating that increasing doses of AG1478 (0.1-1 μM) can inhibit EGF induced phosphorylation of Erk1/2 and Akt, and the IGF induced phosphorylation of Erk1/2, but AG1478 has no effect on IGF-1R activated phosphorylation of Akt. Total Erk1/2 and Akt served as loading controls. D). Western blot analysis demonstrates that treatment with mAb528 only blocks the EGF induced LIP expression, p-Akt, and p-Erk1/2. There is no obvious effect on IGF-1R induced LIP expression, p-Akt, or p-Erk1/2.

To further assess the possibility that EGFR activity may play a role in the IGF-1R stimulated increase in LIP expression, we tested the sensitivity of IGF-1 treated MCF10A cells to the selective EGFR kinase inhibitor, AG1478. Pretreatment of cells for 30 minutes with 0.1, 1 or 5 μM AG1478 prior to addition of 2.6 nM IGF-1 for 16 hr did not inhibit or reduce the IGF-1 mediated increases in LIP expression and did not inhibit the increase in the LIP/LAP ratio (Figure 4A left portion & 4B). As a control, 5 μM AG1478 did lead to the expected decrease in p-EGFR (Figure 4A, lower panel), decreases in EGF mediated LIP expression and the LIP/LAP ratio, and lesser reductions with 0.1 and 1 μM (Figure 4A right portion). Treatment of cells with 0.1, and 1.0 μM AG1478 effectively reduced IGF-1-induced Erk1/2 phosphorylation (Figure 4C left panel) and as expected EGF-induced Erk1/2 phosphorylation (Figure 4C right panel). These data demonstrate that inhibition of EGFR kinase activity reduces IGF-1R mediated Erk1/2 activity and suggest that IGF-1R and EGFR signaling crosstalk in MCF10As to regulate Erk1/2 activity (as suggested in Figure 3B). Our data also demonstrate that inhibition of EGFR signaling with AG1478 does not inhibit IGF-1R induced Akt activity but does block EGF induced Akt activity (Figure 4C). These data are in agreement with published results and demonstrate that IGF-1R mediated Akt activity is not regulated by EGFR signaling, and that IGF-1R mediated Erk1/2 activity is ErbB dependent [26]. IGF-1R mediated Akt activity thus appears to be an important regulator of IGF-1R induced-LIP expression and may also be important for EGF mediated LIP expression.

To validate that IGF-1R induced LIP expression is EGFR independent, we tested an additional EGFR inhibitor. IGF-1R induced LIP expression was not reduced by treatment of MCF10A cells with the EGFR specific, monoclonal antibody, mAb528, which blocks the ligand epitope binding site of EGFR. Although this antibody blockade had no affect on IGF-1R induced LIP expression or the LIP/LAP ratio, it did reduce EGF-induced LIP expression, and the LIP/LAP ratio as expected (Figure 4D). Taken together, these data suggest that although EGFR signaling can crosstalk with IGF-1R signaling, the crosstalk is not required for the IGF-1R mediated regulation of LIP expression in MCF10A cells.

### The role of ERK1/2, and Akt activity in the regulation of IGF-1R induced C/EBPβ-LIP expression

**Figure 5:**
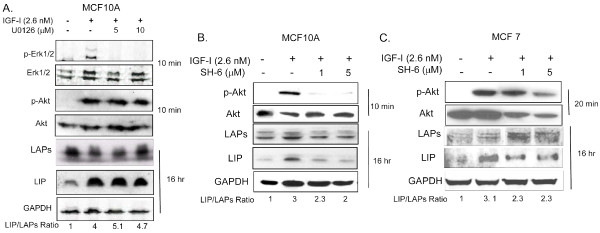
**Akt, but not Erk1/2, is important for IGF-1R mediated regulation of LIP expression**. A). Western blot analysis demonstrating that pretreatment of MCF10A cells with the MEK inhibitor U0126 (5 and 10 μM) blocks IGF-1R activated phosophorylation of Erk1/2, but not phosphorylation of Akt at 10 min. IGF-1R mediated increases in LIP expression were not inhibited by 16 hour of treatment with U0126. Similar results were observed in > 3 independent experiments. B, C). Increasing doses of the Akt inhibitor, SH-6 (1-5 μM), prior to stimulation of cells with IGF-1 (2.6 nM) leads to an inhibition of Akt activity and results in decreased LIP expression at 16 hours in both MCF10A (B) and MCF7 (C) cells. The LIP/LAP ratio is provided.

To better understand the importance of p44/42 MAPK (Erk1/2) and phosphatidylinositol 3-kinase (PI3K)/serine-threonine protein kinase B (Akt) in the regulation of IGF-1R induced LIP expression, cells were pre-treated with a Mek1/2 inhibitor, (U0126), or an Akt inhibitor, (SH-6), 30 minutes prior to stimulation with 2.6 nM IGF-1. As anticipated, 5 and 10 μM U0126 effectively inhibited the IGF-1R induced phosphorylation of Erk1/2 but did not inhibit Akt phosphorylation or the increase observed in LIP expression and the LIP/LAP ratio (Figure 5A). Treatment of MCF10A and MCF7 cells with SH-6, which acts to prevent membrane localization of Akt by competing with Inositol (3, 4, 5) phosphate binding to the Akt pleckstrin homology domain [39], effectively reduced p-Akt expression and LIP expression in IGF-1 treated cells and led to a reduction in the LIP/LAP ratio (Figure 5B, C). Taken together, these results suggest that Akt activity is an important regulator of IGF-1R induced LIP expression.

### C/EBPβ expression is important for cell survival following anoikis

**Figure 6:**
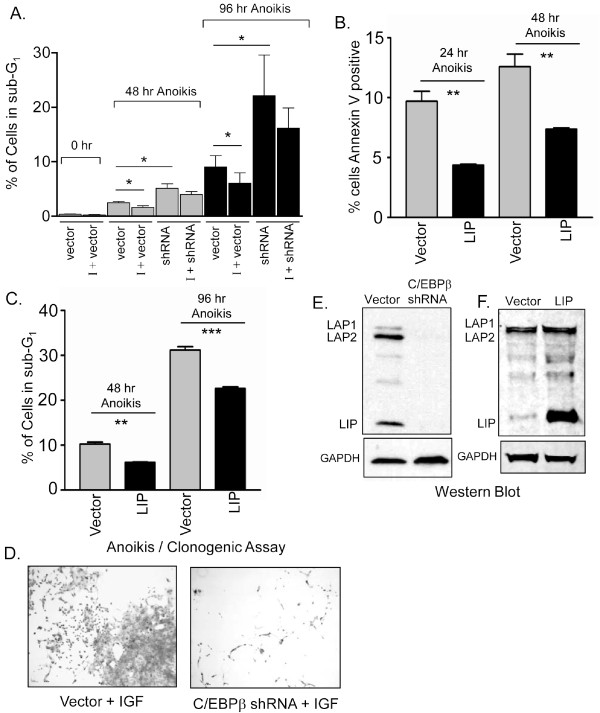
**C/EBPβ expression is important for cell survival following anoikis**. A). Flow cytometry of MCF10A cells in forced suspension culture at 48 hr and 96 hr. Cell death was analyzed by measuring the sub-G_1 _cell cycle fraction. Vector control (Vector) and C/EBPβ knock down (shRNA) cells were treated with Doxycycline (1 μg/ml) for 2 days to activate shRNA expression, followed by one more day of Dox treatment in serum free conditions. IGF-1 (I), (2.6 nM or 39 nM) was then added to the treated cultures at time 0 hour. n = 3B). Flow cytometry of Annexin V positive MCF10A cells, that were transduced to overexpress LIP in forced suspension culture for 24 and 48 hours. n = 3. C). Anoikis assay of MCF10A cells transduced with lenti-LIP (LIP) or vector control (vector) and cultured in suspension for 48 and 96 hours prior to Flow cytometry for the sub G_1 _cell cycle fraction. Statistical significance was determined using an unpaired, 2-tailed T-test with a * p value < 0.05. n = 3. D). After 24 hours of suspension culture in serum free media containing IGF-1 (39 nM), vector control or C/EBPβ knock down cells were transferred to standard 6 well cell culture plates and permitted to adhere and expand for two weeks for analysis of clonogenic outgrowth potential followed by staining with crystal violet. E-F). Western blot analysis of MCF10A cells with shRNA knock down for C/EBPβ (E) or transduced to overexpress LIP (F).

To better understand the biological significance of C/EBPβ expression in response to IGF-1R signaling, we investigated how knock down of C/EBPβ expression affects the well established, anti-apoptotic role of IGF-1R in cell survival. Anoikis, which is an induction of apoptosis that occurs upon loss of cellular adhesion [22], was induced in MCF10A cells via forced suspension culture on low adherence plates for up to 96 hrs, and apoptosis was analyzed as a sub-G1 fraction or Annexin V staining by flowcytometry (Figure 6A, B, C).

Treatment of cells that were serum starved for 24 hrs prior to anoikis, with 39 nM IGF-1, led to an expected increase in cell survival as shown by a significant decrease in apoptosis and reduction in the percent of vector control cells in sub-G_1 _from 2.5% to 1.5% at 48 hr (bars 3 & 4) and from 9% to 6% at 96 hr (bars 7 & 8) of treatment (Figure 6A). Treatment of cells with 2.6 nM IGF-1 led to similar results (data not shown). It is important to note, that before placing IGF-1 treated, vector control cells into the anoikis assay, we checked duplicate plates of cells to validate IGF-1R induced LIP expression. Because the C/EBPβ isoforms are translated from a single mRNA, it is not possible to selectively knock down the individual LIP and LAP isoforms; however successful knockdown of total C/EBPβ expression with shRNA (Figure 6E) led to decreases in cell survival. Increased apoptosis, as observed by the increased number of cells in sub-G_1 _as compared to vector control rose from 2.5% to 5.1% at 48 hr (bars 3 & 5) and 9% to 22% at 96 hr (bars 7 & 9) in the cells with knocked down C/EBPβ expression (Figure 6A). Moreover, in the presence of knocked-down C/EBPβ expression, IGF-1 treatment only moderately increased survival, with decreases in apoptosis from 5.1% to 4% at 48 hr (bars 5 & 6) and 22% to 16% at 96 hr (bars 9 & 10). These decreases in apoptosis were not statistically significant.

Because we have demonstrated in this study that IGF-1R signaling increases LIP expression and the ratio of LIP/LAP, we sought to test the effects of LIP overexpression on survival from anoikis, in a manner similar to that described in Figure 6A. Overexpression of LIP in MCF10A cells was accomplished using a pEIZ (HIV-Zsgreen) lentiviral construct driven by the EF-alpha 1 promoter [40]. Overexpression of LIP (Figure 6F) led to decreases in apoptosis as evidenced by the number of Annexin V positive cells (Figure 6B) and the accumulation of cells in sub-G1 at both 48 hr and 96 hr of anoikis (Figure 6C). These data suggest that the LIP isoform has an anti-apoptotic action and plays a role in cellular survival of anoikis. Thus the biological consequence of IGF-1R mediated increases in LIP expression may include the actions of LIP to participate in the regulation of cell survival. Our data demonstrate that treatment of cells with IGF-1 or overexpression of LIP leads to decreases in the percentage of cells in sub-G1, and decreases in the number of cells positive for Annexin V, thus representing a decrease in apoptosis (Figure 6A, B, C).

Taken together, the data in Figure 6 demonstrate that C/EBPβ knockdown leads to increased cell death and an accumulation of cells in sub-G_1 _and suggest that C/EBPβ expression is important for survival and resistance to anoikis. Furthermore, we showed that IGF-1R treatment can partially rescue control cells from anoikis; however, cells with reduced C/EBPβ expression, are not successfully rescued from anoikis. This is most clearly observed in clonogenic outgrowth assays of C/EBPβ knock-down cells (Figure 6D). Suspension culture of vector control and C/EBPβ knock-down cells, in the presence of IGF-1 for 24 hr, followed by harvest and subsequent plating for adherent growth revealed a dramatic reduction in the survival and clonogenic activity of cells with knocked down C/EBPβ expression (Figure 6D). Similarly, overexpression of LIP reduced anoikis, as evidenced by the decreased number of Annexin V positive cells and the decreased number of sub G1 cells. In summary, C/EBPβ expression appears to play an important role in protection from anoikis and may be an integral downstream mediator of the protective effects of IGF-1R signaling.

In summary, our data demonstrate that IGF-1 stimulation of mammary epithelial cells leads to increased expression of LIP and an elevation in the LIP/LAP ratio. We additionally demonstrate that IGF-1R induced-LIP expression is biologically active as determined on a C/EBP responsive promoter construct. Although IGF-1R signaling can crosstalk with EGFR signaling to regulate Erk1/2 activity in our study, IGF-1R induced LIP expression is independent of EGFR signaling. We demonstrate that Akt activity is a critical determinant in the regulation of IGF-1R induced LIP expression and that EGFR-dependent, Erk1/2 activity is not necessary for IGF-1R induced LIP expression. Lastly we show that LIP plays a role to increase the survival of cells from anoikis and may participate in IGF-1R mediated suppression of anoikis.

## Discussion

Our data, as well as that from others, suggest that oncogenic signaling pathways such as IGF-1R, EGFR [32], and ErbB2 [33] regulate increases in LIP expression and the LIP/LAP ratio. IGF-1R, EGFR and ErbB2 and are also critical regulators of tumorigenesis and can regulate cellular survival of anoikis [34,41]. IGF-1R signaling is known to play an important role in the resistance of cells to apoptosis and this anti-apoptotic effect is most strongly observed during anchorage-independent conditions (reviewed in [42] and in metastatic breast cancer cells [43,44] The survival of cells in suspension, or the ability to suppress anoikis, is a critical step in the progression of invasive cancer because metastatic cells must survive under anchorage-independent conditions as they move from the primary tumor to distant sites. The molecular mechanisms that regulate anoikis in invasive cancer cells are poorly understood, but we have demonstrated that loss of C/EBPβ expression renders cells more susceptible to anoikis, even in the presence of IGF-1R signaling. Moreover, LIP overexpression protects cells from anoikis. Our study is the first to document a role for C/EBPβ in the survival of mammary epithelial cells under anchorage-independent growth conditions. The biological significance of elevated LIP expression as a consequence of IGF-1R receptor signaling holds important implications for the LIP/LAP ratio as a critical mediator of anchorage-independent growth in breast cancer.

Taken together, C/EBPβ-LIP appears to be an important downstream target for EGFR, ErbB2 and IGF-1R signaling, and particpates in the regulation cell survival and apoptosis. This survival mechanism may actually be quite universal and not unique to breast cells. For example, macrophages require C/EBPβ for survival in response to Myc/Raf transformation [25] and hepatic stellate cells that are DNA damaged via CCl4 induced free radical formation [23] also need C/EBPβ for survival. In keratinocytes that have suffered DNA damage, C/EBPβ promotes cell survival by reducing p53 expression and activity [45]. Reduced levels of C/EBPβ can thereby sensitize cells to apoptosis and this has been observed both in our anoikis model (Figure 6A) and in C/EBPβ null mice which display resistance to DMBA-induced skin tumorigenesis [24].

Numerous parallels exist between the biological effects of IGF-1R signaling and that of LIP overexpression. For instance, both the IGF-1/insulin receptor families and the C/EBPβ isoforms play important roles in cellular processes that regulate mammary development and breast cancer such as cell cycle control, proliferation, and differentiation. As an example, cell cycle entry and progression to the restriction point in late G_1 _is controlled by growth factors, such as IGF-1; however the C/EBPβ isoforms also interact with or regulate similar cell cycle proteins such as p53 [46], Rb [47,48] CDK2, cyclin A, cyclin E [49] cyclin D1 [50] p21Cip1, [51], and p15INK4b [15].

In regards to development, inhibition of IGF-1R signaling or knockdown of C/EBPβ expression disrupts mammary gland development. For example, mammary gland development is restricted in both IGF-1 null mice [52] and in IGF-1R-null mice [53]. Similar phenotypes are observed in the C/EBPβ null mouse, where deletion of the C/EBPβ isoforms leads to defective mammary gland development and reduced milk production [1,2]. Conversely, the activation or elevation of IGF-1R or LIP expression induces mammary proliferation and tumorigenesis. For example, overexpression of IGF-1R in the mouse mammary gland leads to tumorigenesis [54-58] while in a similar fashion, transgenic expression of LIP in mouse mammary glands induces hyperproliferation and tumorigenesis [12].

Moreover, in women, elevated LIP or IGF-1R expression are independently associated with breast cancer. Approximately 23% of aggressive breast cancers contain elevated LIP and this increase in LIP is associated with reduced estrogen and progesterone receptor expression and an otherwise poor prognosis [13]. Both the IGF-1R and insulin receptor are activated and expressed at elevated levels in breast cancer [16,59]. In fact, patients with type 2 diabetes mellitus are suspected to be at increased risk of developing breast cancer [60]. When considering the fact that LIP expression is regulated by IGF-1R signaling, and that numerous biological similarities exist between LIP overexpression and IGF-1R signaling, one can only speculate that LIP may in part, be a critical mediator of many of the downstream effects of IGF-1R signaling

Although our study focused on the IGF-1R regulation of LIP and LAP expression; the reverse has also been observed, and IGF-1 expression and/or activity has been shown to be regulated by the LIP and LAP isoforms in macrophages, hepatocytes, and osteoblasts (Reviewed in [4]). With the exception of our current study in the mammary epithelial cell line MCF10A, little is known about IGF-1 and LIP/LAP interactions in breast epithelial cells. In bone marrow derived macrophages isolated from the C/EBPβ K/O mouse, IGF-1 expression is moderately decreased in response to the loss of C/EBPβ expression [25]. Similarly, in hepatocytes, the addition of C/EBPβ-LAP in the human hepatoma cell line Hep3B increases IGF-1 expression [61]. Overexpression of LIP alone appears to have no effect on IGF-1 promoter activity, but does abolish the transactivation induced by LAP [61]. Moreover, C/EBPβ is believed to play a role in the proliferation and differentiation of osteoblasts via regulation of IGF-1 and studies have shown that the protein levels and DNA binding activity of the C/EBPβ isoforms, LAP1, LAP2 and LIP are elevated in proliferating osteoblasts (MC3T3-E1 cells) and down regulated upon differentiation [62]. In light of these studies and our recent data, we speculate that the C/EBPβ-LIP and LAP isoforms participate in a feedback loop to regulate IGF-1 signaling; however, this hypothesis will require further experimentation.

## Conclusions

Previously we demonstrated in MCF10As that EGFR signaling increases expression of the C/EBPβ-LIP isoform and that this regulation is dependent upon Erk1/2 activity [32]. We now show that IGF-1 and insulin signaling regulate LIP expression in MCF10A cells, and that Akt activity, rather than Erk1/2 is a critical determinant for IGF-1R induced LIP expression. In some cellular contexts, cross talk has been shown to occur between the IGF-1 receptor and the EGF receptor (EGFR) during mediation of IGF-1 signaling [26,27,29,63]. The mechanism of crosstalk may involve the IGF-1 stimulated cleavage and solubilization of EGFR pro-ligands which lead to EGFR activation [26] or the direct interaction of IGF-1R with EGFR to form EGFR-IGF-1R hetero-oligomers [29]. Regardless of the mechanism at work in our study, crosstalk between IGF-1 and EGFR is not necessary for the regulation of LIP expression by IGF-1. The reasons for this may be explained by the observation that PI3K/Akt pathway and Ras/Erk1/2 pathways downstream of IGF-1 signaling are often functionally dissociated [26,29]. IGF-1 induced Erk1/2 activity can be predominantly activated by the transactivation of EGFR in response to IGF-1 while Akt activation is independent of EGFR activity [26,29]. Our data clearly show that IGF-1 mediated increases in LIP expression are not regulated by EGFR dependent Erk1/2 activity, but rather by IGF-1 induced Akt activity. The mechanism by which Akt activates LIP translation and expression remain to be elucidated.

## Methods

### Cell Culture

Cultured mammary epithelial cells, MCF10A, were grown in Dulbecco modified Eagle medium (DMEM)-F12 (Invitrogen, USA) supplemented with 5% donor horse serum (Invitrogen, USA), 20 ng/ml of recombinant human EGF (Invitrogen, USA), 10 μg/ml of bovine pancreatic insulin (Sigma, USA), 100 ng/ml of cholera toxin (Sigma, USA), 0.5 μg/ml of hydrocortisone (Sigma, USA), and 5 μg/ml of gentamycin sulfate (Invitrogen, USA). MCF7 cells were grown in Eagle's Minimum Essential Medium (MEM) (Mediatech, USA) supplemented with 0.01 mg/ml bovine insulin and 10% fetal bovine serum (Hyclone, USA). C/EBPβ null cells were culture in Hepes buffered, Dulbecco modified Eagle medium (DMEM)-F12 (Invitrogen, USA) supplemented with 2% adult bovine serum (Invitrogen, USA), 5 ng/ml of recombinant human EGF (Invitrogen, USA), 10 μg/ml of bovine pancreatic insulin (Sigma, USA) and 5 μg/ml gentamycin sulfate.

### Suspension Culture/Anoikis Assay

To knock down C/EBPβ expression, C/EBPβ (Tet-On) and control TRIPZ™lentiviral shRNAmir constructs (Open Biosystem, USA) were stably transduced into MCF-10A cells by infection and puromycin selection. Prior to suspension culture, the cells were treated with Doxycycline (1 μg/ml) for 2 days to activate shRNA expression, followed by one more day of Dox treatment in serum free conditions to synchronize the cells and to generate a maximal knockdown of C/EBPβ expression. To prevent adherence, cells were transferred to Costar 6 well ultra low attachment plates (20,000 cells/well) or to 1% agar coated plates (500,000 cells/10 cm dish) for 24, 48 and 96 hrs in the presence or absence of IGF-1 (2.6 nM and 39 nM). After 24 hrs, suspended cells were transferred to standard 6 well cell culture plates and permitted to adhere to analyze survival via clonogenic outgrowth for two weeks followed by staining with crystal violet. Flow cytometry was conducted on cells collected at 48 and 96 hrs of suspension culture. Briefly, suspended cells were collected by centrifuge at 1000 rpm for 5 min. To prevent clustering, cells were digested in 1× trypsin at 37°C for 5 min, followed by washing with HBSS. Cells were then resuspended in (0.6% NP-40, 3.7% Formalin, 11% Hoechst 33258 in PBS) for Flow cytometry. Cell death was analyzed by measuring the sub-G_1 _cell cycle fraction. LIP was overexpressed in MCF-10A cells using a pEIZ (HIV-Zsgreen) lentiviral construct driven by the EF-alpha 1 promoter (kindly provided by Dr. Zena Werb [40]) and cells were sorted. Annexin V-PE Apoptosis detection kit was purchased from BD Biosciences and performed according to manufacturer's instructions.

### Cell Treatment, Protein Isolation and ECL Western Blot Analysis

MCF10A and MCF7cells were plated at a density of 1.7 × 10^6^/100 mm and upon reaching 75 to 80% confluency, the growth medium was removed and replaced with a serum-free, defined medium containing DMEM-F12, 100 ng/ml cholera toxin, 0.5 μg/ml of hydrocortisone, and 5 μg/ml of gentamycin sulfate for MCF10A, and MEM for MCF7. Cells were maintained in defined medium for 24 hour prior to the addition of ligand: human EGF (Invitrogen, USA), IGF-1 (Sigma, USA), insulin (Sigma, USA) and harvested at 10-20 min or 16 hr after the addition of ligand. The MEK inhibitor, U0126 (Calbiochem, USA), the Akt inhibitor, SH-6 (Axxora Platform, USA), the EGFR inhibitor, AG1478 (Sigma, USA), and the blocking antibody EGFR-mAb528 (Santa Cruz Biotechnology, USA) were added 30-60 min before addition of ligand. Cells harvested at 16 hr were sonicated in radioimmunoprecipitation assay (RIPA) buffer (50 mM Tris-HCl [pH 7.4], 1% NP-40, 0.25% deoxycholate, 150 mM NaCl, 10 mM EGTA, 0.2% sodium dodecyl sulfate [SDS]) containing a protease inhibitor cocktail (Sigma, USA) and a phosphatase inhibitor I and II mixture (Sigma, USA). Aliquots of the lysates containing 100-200 μg of protein were boiled at 100°C for 10 min, electrophoresed on denaturing SDS-7% or 12% polyacrylamide minigels, and then transferred to polyvinylidene difluoride membranes (PVDF, Millipore, Bedford, Mass. USA). Blots were blocked 1-2 hr in TBST (20 mM Tris [pH 7.5], 150 mM NaCl, 0.5% Tween 20) containing 5% Carnation dry milk and then incubated with primary antibody for 1-2 hr (or overnight for antibodies directed against phospho-proteins) in TBST-1-5% carnation milk. Primary antibodies used were monoclonal and polyclonal anti-C/EBPβ (1:250, Santa Cruz, USA), polyclonal anti-GAPDH (1:5000, Trevigen, Gaithersburg, MD, USA), polyclonal β-actin (1:1000 Santa Cruz, USA), monoclonal anti-phospho-p44/42 (1:2000, Cell Signaling, Beverly, MA, USA), polyclonal anti-p44/42 (1:2000, Cell Signaling, USA), monoclonal anti-phospho Akt, polyclonal Akt (1:1000, Cell Signaling, USA), polyclonal anti-EGFR (1:1000 Santa Cruz, USA), monoclonal anti-phospho-EGFR (Tyr 845, 1:1000, Cell Signaling, USA). Blots were washed with TBST three times for 5 to 10 min each with agitation and then incubated for 1 hr with either goat anti-mouse-horseradish peroxidase (HRP) conjugate (Santa Cruz. USA) or goat-anti-rabbit-HRP (Bio-Rad, Hercules, CA, USA) in TBST-1-5% carnation. Proteins were visualized by either DURA or FEMTO chemiluminescence (Super Signal; Pierce, Rockford, Ill. USA) and HyBlot CL film (Denville Scientific, Metuchen, NJ, USA). Blots were stripped in Re-blot Plus Mild Solution (Chemicon, Temecula, CA, USA) for reprobing.

### Western Blot Analysis Using Odyssey Infrared Imaging

Proteins were electrophoresed and transferred to PVDF membranes as described above. Membranes were blocked for 1 hr in Odyssey blocking buffer. Primary antibodies (1:250, monoclonal anti-C/EBPβ, Santa Cruz, USA), polyclonal anti-GAPDH (1:5000, Trevigen, Gaithersburg, MD, USA) and secondary antibodies (1:5000, goat anti-mouse IR Dye 800 CW, LI-COR Biosciences and 1:5000, goat anti-mouse IR Dye 680 DX, LI-COR, USA) were diluted in blocking buffer with 0.1% Tween-20 and incubated with the blot for 1 hr at room temperature. After washing, the membranes were scanned using Li-COR's Odyssey infrared imaging system and quantitated using Odyssey 3 software.

### Quantitative Realtime PCR

MCF10A and MCF7cells were plated at a density of 75 to 80% confluency, the growth medium was removed and replaced with a serum-free, defined mediums as described. Cells were maintained in defined medium for 24 hour prior to the addition of human IGF-1 (Sigma, USA) and harvested at 16 hr after the addition of ligand by adding 1 ml Trizol (Invitrogen, USA). Total RNA was extracted according to the manufacturer's instruction. First-strand cDNA was prepared with 5 μg total RNA, random primers and reverse transcriptase (SuperScript II RNase H, Invitrogen, USA) according to the manufacturer's instruction. Quantitative PCR was performed by using real-time PCR iCycler (Bio-Rad, USA). PCR reaction and C/EBPβ primers were: sense 5' AACTCTCTGCTTCTCCCTCTG 3'; antisense 5'AAGCCCGTAGGAACATCTTT 3'. Ct values were converted to relative expression using the delta-delta Ct method, allowing normalization to both 18S and untreated control. The primer sequences for 18S were sense 5' GTAACCCGTTGAACCCCATTC 3'; antisense: CCATCCAATCGGTAGTAGCG 3'.

### Luciferase Assay

To validate the activity of individual LIP and LAP2 constructs, a C/EBP consensus luciferase construct (500 ng) and a Renilla construct (20 ng) as internal control were cotransfected with LAP2 and LIP individually or together at different ratios into C/EBPβ null cells to a total of 2500 ng plasmid DNA. Control vector serves as both a control for basal activity and to match the quantity of plasmid DNA. Luciferase and Renilla activities were recorded at 48 hrs. For the IGF experiment, MCF-10A cells were cultured in Falcon 24-well plates and at 70% confluency, were transfected with a C/EBP consensus Luciferase construct (500 ng) and a Renilla construct (20 ng) as internal control. Transfection was conducted using Fugene reagent (Roche, Switzerland, according to manufacturer instructions) and cells were maintained in serum free medium for 24 hrs. The cells were then treated with 2.6 nM IGF-1 for 16 hrs in serum free medium and luciferase activity was analyzed at the end of treatment. The relative luciferase activity was calculated as Luciferase value/Renilla value. n = 5

### Immunoprecipitation and Immuno-Blot Analysis of EGFR

MCF10A cells incubated with ligand for 10 min were extracted in RIPA buffer without SDS, and sonicated. Protein extracts (1 mg) were pre-cleared for 1 hr at 4°C with protein G-PLUS agarose (Santa Cruz, USA), then immunoprecipitated overnight at 4°C with anti-EGFR (1:1000, Santa Cruz, USA) or 4G10-conjugated agarose beads (Upstate Biotechnology, Waltham, MA, USA) to immunoprecipitate IGF-1R/IR. The beads were rinsed 3 times with RIPA (without SDS), sample buffer was added, the mixture boiled for 10 minutes followed by electrophoresis through SDS-7% polyacrylamide minigels, and transfer to PVDF. Immuno-blots were performed as above using anti-phospho-EGFR (1:1000, Cell Signaling, USA), anti-IR (1:1000 Upstate Biotechnology, USA) or anti-IGF-1R (1:1000 Upstate Biotechnology, USA).

## Competing interests

The authors declare that they have no competing interests.

## Authors' contributions

HLL carried out the LIP/LAP ratio experiments, the anoikis studies, some of the inhibitor cell signaling studies and conducted the ErbB analysis. BRB generated the initial data which showed that IGF-1 and insulin regulates LIP expression and conducted some of the inhibitor cell signaling studies. CAZ participated in the design of all experiments, interpreted the data, conducted the statistical analyses and wrote the manuscript. All authors read and approved the final manuscript.
